# Chronic Food Antigen-specific IgG-mediated Hypersensitivity Reaction as A Risk Factor for Adolescent Depressive Disorder

**DOI:** 10.1016/j.gpb.2019.05.002

**Published:** 2019-06-21

**Authors:** Ran Tao, Zhicheng Fu, Lijun Xiao

**Affiliations:** 1 Department of Psychological Medicine, The Seventh Medical Center of Chinese PLA General Hospital, Beijing 100700, China; 2 Department of Trace Element Nutrition, National Institute for Nutrition and Health, Chinese Center for Disease Control and Prevention, Beijing 100050, China

**Keywords:** Major depressive disorder, Blood–brain barrier, Histamine, Hypersensitivity reaction, Inflammation

## Abstract

**Major depressive disorder** (MDD) is the most common nonfatal disease burden worldwide. Systemic chronic low-grade **inflammation** has been reported to be associated with MDD progression by affecting monoaminergic and glutamatergic neurotransmission. However, whether various proinflammatory cytokines are abnormally elevated before the first episode of depression is still largely unclear. Here, we evaluated 184 adolescent patients who were experiencing their first episode of depressive disorder, and the same number of healthy individuals was included as controls. We tested the serum levels of high-sensitivity C-reactive protein (hs-CRP), tumor necrosis factor-α (TNF-α), IgE, 14 different types of food antigen-specific IgG, **histamine**, homocysteine, S100 calcium-binding protein B, and diamine oxidase. We were not able to find any significant differences in the serum levels of hs-CRP or TNF-α between the two groups. However, the histamine level of the patients (12.35 μM) was significantly higher than that of the controls (9.73 μM, *P* < 0.001, Mann–Whitney *U* test). Moreover, significantly higher serum food antigen-specific IgG positive rates were also found in the patient group. Furthermore, over 80% of patients exhibited prolonged food intolerance with elevated levels of serum histamine, leading to hyperpermeability of the **blood–brain barrier**, which has previously been implicated in the pathogenesis of MDD. Hence, prolonged high levels of serum histamine could be a risk factor for depressive disorders, and antihistamine release might represent a novel therapeutic strategy for depression treatment.

## Introduction

Major depressive disorder (MDD) is a kind of mental disease that affected approximately 216 million people (3% of the world's population) in 2015 and that is projected to have the second-highest global burden of disease in all age groups and sexes by 2020 [1]. The present evidence suggests that many factors are involved in the pathophysiology of MDD, including heredity, neurotransmitters, immunity, oxidation, and the inflammatory system [2], [3]. However, the pathological mechanism of MDD is not yet clear. In the field of neurochemistry, the most widely accepted hypothesis of MDD is the lack of monoamine neurotransmitters, especially 5-hydroxytryptamine (5-HT) neurotransmitters [4].

Previous findings have shown that at least some subtypes of depression are associated with chronic low-grade systemic inflammation in adults [5], [6]. The levels of serum inflammatory markers are increased in some adult MDD patients. For example, the level of C-reactive protein (CRP), which reflects the state of systemic inflammation, is elevated in the serum of at least 30% of MDD patients [7], [8]. Serum levels of proinflammatory cytokines, such as tumor necrosis factor-α (TNF-α), are also higher in patients with depression than in those without depression or anxiety [9], [10], [11]. Diseases related to chronic inflammation, such as cardiovascular diseases, inflammatory bowel diseases, and rheumatoid diseases, are associated with a high risk of MDD [12], [13].

Many previous studies have indicated that proinflammatory cytokines can disrupt neurotransmitter metabolism affect neuronal transmission [14], [15], [16], and eventually trigger corresponding psychiatric symptoms. These studies have established a link between depression and inflammation. However, the role of inflammation in the occurrence and development of depression is still unclear, mainly because the existing studies have been conducted on adult patients, and many factors, such as chronic diseases, substance addiction, or social environment, may interfere with the observed endpoints. It has been reported that adult depression often arises from adolescent depression, so the study of adolescent depression can reflect the early mechanisms of MDD development [17]. It is necessary to determine whether there is a causal relationship between systemic inflammation and depressive disorder by including adolescent depressive patients as the subjects.

In addition to inflammation, the blood–brain barrier (BBB) is also closely associated with MDD and other mental diseases such as Alzheimer's disease and Parkinson's disease [18], [19], [20]. The BBB is a highly selective semi-permeable barrier, formed by close connections of special vascular endothelial cells. The BBB is the main barrier of the central nervous system (CNS) and prevents the influx of active substances from the peripheral circulation system [21]. The high permeability of the BBB can cause the influx of some risk factors, such as toxins, pathogenic substances, and proinflammatory cytokines from the peripheral circulation system. The influx of these factors has been confirmed to result in the inflammation of neurons in the brain, disturbing neurotransmitter metabolism and function [19]. Therefore, at the early stage of depressive disorders, identifying the factors that induce the high permeability of the BBB would be helpful in determining whether BBB leakage is related to the onset of depression. Histamine has been shown to be one of the main factors that induces high BBB permeability [18]. Therefore, our present study focuses on histamine and the related factors that affect histamine metabolism (IgG and IgE) and their effects on substances of cell metabolism.

## Results

In this study, we recruited adolescent depressive patient with the age-matched healthy subjects as control. The inclusion criteria for subject enrollment were described in Materials and methods section. The adolescent depressive disorder patient (ADP) group consisted of 184 patients with moderate depression, including 114 males and 70 females, with average age of 17.2 ± 1.76 years old. The control normal adolescent student (NAS) group consisted of 184 students, including 105 males and 79 females, with average age of 17.4 ± 1.58 years old. There were no significant differences in sex and age between the patient and control groups (*P* > 0.05, Chi-square test).

### Higher levels of serum histamine were found in the ADP group

**Figure 1:**
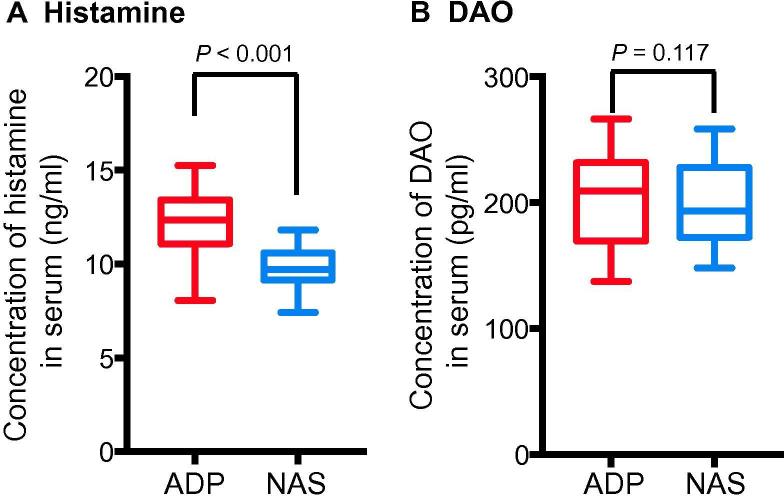
**Adolescent patients with depressive disorder have significantly-elevated serum histamine levels** Serum levels of histamine (**A**) and DAO (**B**) were quantified in both the ADP and NAS groups. Data are shown as median (quartile spacing) [M (P25, P75)] (*N* = 184). *P* values were determined using Mann–Whitney *U* test and differences with *P* < 0.05 are considered significant. DAO, diamine oxidase; ADP, adolescent depressive disorder patient; NAS, normal adolescent student.

Histamine is a kind of organic nitrogen-containing compound that participates in the immune response. Histamine receptors, including H1, H2, and H3, are all expressed in the CNS. These receptors are involved in the release of acetylcholine, norepinephrine, 5-HT, and other neurotransmitters [22]. Some studies have shown that high levels of histamine are found in the cerebrospinal fluid and brain parenchyma of individuals with various neurodegenerative diseases [23], [24], [25]. However, these studies did not confirm the general level of histamine. We compared the serum histamine levels between the ADP group and the NAS group. Among the 184 patients, the average concentrations of histamine in the ADP group and the NAS group were 12.35 (11.11, 13.42) ng/ml and 9.73 (9.16, 10.6) ng/ml, respectively (*P* < 0.001, Mann–Whitney *U* test) (Figure 1A). To determine whether the elevated serum histamine level was associated with an abnormal pathway of histamine degradation, we examined the level of histamine metabolizing enzyme diamine oxidase (DAO) in these two groups, and no significant difference was found. The mean concentrations of DAO were 209.24 (169.50, 231.91) pg/ml and 193.64 (172.45, 225.12) pg/ml for ADP and NAS groups, respectively (*P* = 0.117) (Figure 1B). These results indicate that the elevated level of serum histamine is not the result of a histamine-related metabolic disorder, suggesting that histamine may be involved in the pathogenetic process of adolescent depressive disorder.

### The ADP group exhibited higher positive rate of IgE and food antigen-specific IgG

Histamine is mainly stored in labrocytes and basophils, and it is released when IgE bound to allergens interacts with IgE receptors on labrocytes and basophils. A recent study shows that in addition to IgE receptors, IgG receptors are also expressed on the surface of labrocytes and basophils [26], [27]. In addition, in animal studies, antigen-bound IgG immune complexes have also been shown to induce high levels of serum histamine [28]. To investigate whether high levels of serum histamines are caused by IgE or IgG, we examined the level of IgE and 14 types of food antigen-specific IgG in serum samples from the two groups (Table 1). Among the 184 patients in the ADP group, 66 (35.87%) patients had high IgE (>100 KU/l), whereas the corresponding number is 42 (22.83%) in the NAS group (*P* < 0.001, Chi-square test). The average IgE concentrations of the ADP and NAS groups were 49.80 (10.00, 413.98) KU/l and 31.63 (10.00, 88.50) KU/l (*P* < 0.001, Mann–Whitney *U* test), respectively. The high positive rate of IgE in the ADP group suggests that IgE-mediated type I hypersensitivity may be involved in the elevated level of serum histamine in adolescent patients with depression.

**Table 1:** **Serum IgE and food antigen-specific IgG levels in the ADP and NAS groups**

	**ADP (*n* = 184)**	**NAS (*n* = 184)**	***P* value**
IgE (KU/l)	49.8 (IQR: 10.0–414.0)	31.6 (IQR: 10.0–88.5)	< 0.001
Percentage (No.) of subjects with high IgE	35.87% (66)	22.83% (42)	0.006
Percentage (No.) of subjects positive for food antigen-specific IgG	89.67% (165)	13.04% (24)	< 0.001
Egg	75% (138)	11.96% (22)	< 0.001
Milk	47.28% (87)	10.33% (19)	< 0.001
Soybean	15.22% (28)	6.52% (12)	0.007
Wheat	13.59% (25)	8.15% (15)	0.094
Rice	11.96% (22)	1.63% (3)	< 0.001
Tomato	11.96% (22)	1.63% (3)	< 0.001
Codfish	11.41% (21)	5.43% (10)	0.039
Crab	8.15% (15)	2.17% (4)	0.01
Corn	8.15% (15)	1.09% (2)	0.001
Mushroom	7.61% (14)	1.63% (3)	0.006
Shrimp	6.52% (12)	2.72% (5)	0.082
Pork	3.26% (6)	2.17% (4)	0.521
Chicken	2.72% (5)	0.54% (1)	0.01
Beef	0	0	-

*Note*: Subjects with serum IgE levels higher than 100 KU/l are considered to have high levels of IgE, whereas subjects with serum IgG levels higher than 50 KU/l are considered to be positive for food antigen-specific IgG. *P* values were determined using Chi-square test or Fisher’s exact test and differences with *P* < 0.05 are considered significant. ADP, adolescent depressive disorder patient; NAS, normal adolescent student; IQR, interquartile range; IgE, Immunoglobulin E.

The positive rate of food antigen-specific IgG was 89.67% (165/184) in the ADP group and 13.04% (24/184) in the NAS group (*P* < 0.001, Chi-square test; Table 1). All the 14 types of food examined in this study are commonly consumed by the Chinese people. Chronic contact with these food antigens might induce a higher food antigen-specific IgG-mediated type III hypersensitivity reaction state in the ADP group. Overall, the positive rate of food antigen-specific IgG was higher in the ADP group, suggesting that IgG-mediated type III hypersensitivity may be primarily responsible for the elevated serum histamine level in adolescent patients with depression.

### The serum S100B levels were higher in the ADP group

**Figure 2:**
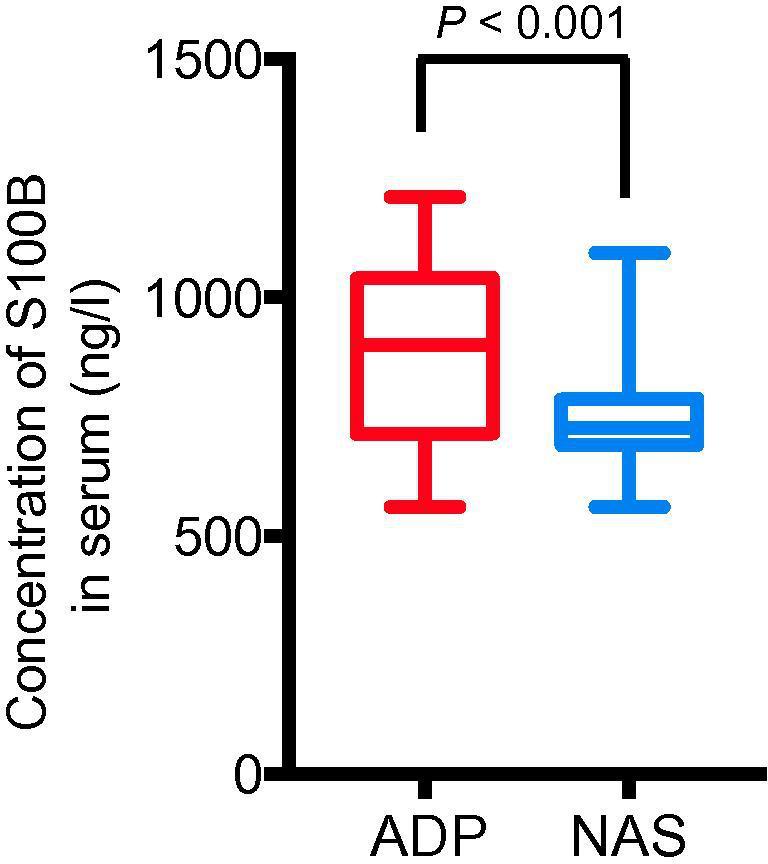
**Adolescent patients with depressive disorder have significantly**-**elevated serum S100B levels** Serum levels of S100B were quantified in both the ADP and NAS groups. Data are shown as median (quartile spacing) [M (P25, P75)] (*N* = 184). *P* values were determined using Mann–Whitney *U* test and differences with *P* < 0.05 are considered significant. S100B, S100 calcium-binding protein B.

Normally, histamine does not pass through the BBB, however, long-term high level of serum histamine has been confirmed to cause high permeability of the BBB [18]. The finding that the serum histamine level was higher in the ADP group prompted us to determine whether or not the elevated serum histamine level in ADP group is associated with BBB leakage. S100 calcium-binding protein B (S100B), which is mainly expressed in the astrocytes of the CNS, has been considered a biomarker of BBB leakage. We thus measured the level of serum S100B in these two groups. We found that the average concentration of serum S100B in the ADP group [901.97 (713.84, 1039.07) ng/l] was significantly higher than that in the NAS group [725.17(691.17, 786.37) ng/l] (*P* < 0.001, Mann–Whitney *U* test) (Figure 2). Therefore, the enhanced BBB permeability in the ADP group is probably attributed to the elevated serum histamine level.

### Higher homocysteine levels were detected in the ADP group

**Figure 3:**
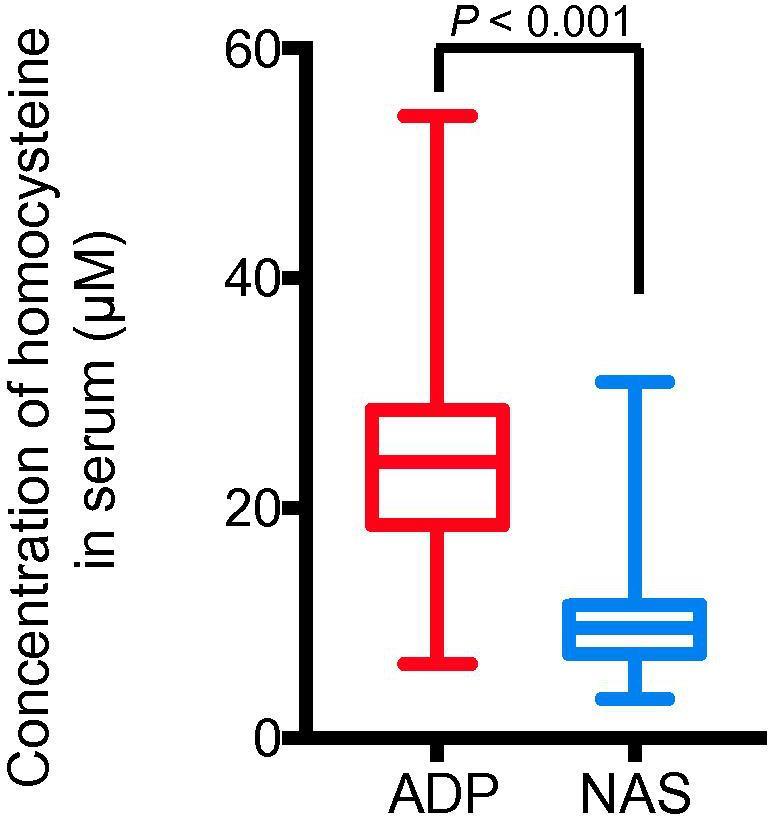
**Adolescent patients with depressive disorder have significantly**-**elevated serum homocysteine levels** Serum levels of homocysteine were quantified in both the ADP and NAS groups. Data are shown as median (quartile spacing) [M (P25, P75)] (*N* = 184). *P* values were determined using Mann–Whitney *U* test and differences with *P* < 0.05 are considered significant.

Homocysteine is a sulfur-containing amino acid, which is mainly affected by nutritional deficiency due to lower B vitamin intake and worse intestinal absorption. The metabolism of homocysteine requires the participation of vitamin B6, vitamin B12, folic acid, and other substances [29]. If an individual is deficient in the substances above, the concentration of homocysteine in the blood would increase [30]. We found that the average concentration of homocysteine in the ADP group [24.00 (18.5, 28.45) μM] was significantly higher than that in the NAS group [9.55 (7.45, 11.53) μM] (*P* < 0.001, Mann–Whitney *U* test) (Figure 3). These data suggest that the increase in homocysteine concentration may be due to the reduced intestinal absorption caused by hypersensitivity mediated by chronic food antigen-specific IgG and consequently the deficiency of vitamin B.

### There was no significant difference in systemic inflammatory markers between ADP and NAS groups

**Figure 4:**
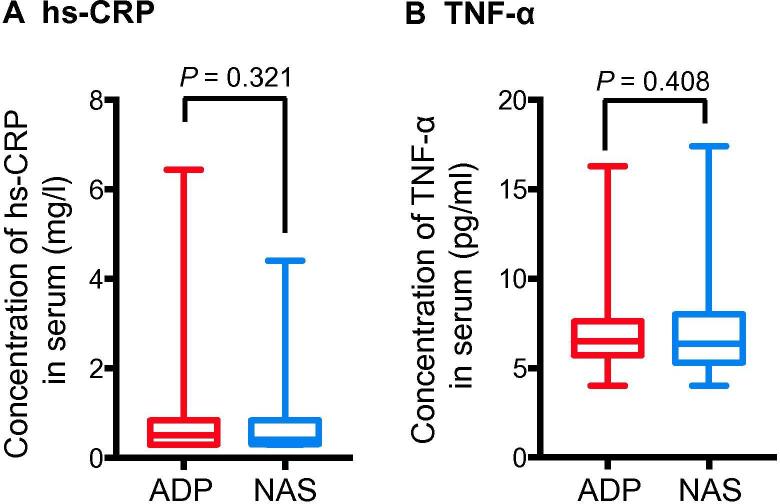
**Adolescent patients with depressive disorder and the normal adolescent students have comparable levels of serum inflammation markers** Serum levels of hs-CRP (**A**) and TNF-α (**B**) were quantified in both the ADP and NAS groups. Data are shown as median (quartile spacing) [M (P25, P75)] (*N* = 184). *P* values were determined using Mann–Whitney’s *U* test and differences with *P* < 0.05 are considered significant. hs-CRP, high-sensitivity C-reactive protein; TNF-α, tumor necrosis factor-α.

Clinical studies have reported that systemic inflammation plays an important role in the occurrence and development of depression [5], [6]. To determine whether chronic systemic inflammation plays a role in the pathogenesis of depression, we compared the serum levels of systemic inflammation biomarkers, *i.e.*, high-sensitivity C-reactive protein (hs-CRP) and tumor necrosis factor-α (TNF-α), between these two groups. We found that 37/184 (20.11%) and 34/184 (18.48%) of the participants had hs-CRP at the concentration >1 mg/l, with average concentrations of 0.50 (0.30, 0.84) mg/l and 0.41 (0.32, 0.84) mg/l (*P* = 0.321, Mann–Whitney *U* test) in the ADP and NAS groups, respectively. Regarding TNF-α, 36/184 of the patients in the ADP group and 39/184 of the healthy controls in the NAS group had an elevated level (>8.1 pg/ml), with average concentrations of 6.5 (5.7, 7.6) pg/ml and 6.35 (5.3, 8.0) pg/ml, respectively (*P* = 0.408, Mann–Whitney *U* test) (Figure 4). Therefore, we did not observe significant difference in the concentrations of the two aforementioned biomarkers between ADP and NAS groups, suggesting that the pathogenesis of depression may not be attributed to systemic inflammation.

## Discussion

Although chronic low-grade systematic inflammation has been implicated in the progression of some subtypes of depression in adults [5], [6], its association with the first-episode adolescent depression is still largely unknown. In this study, we recruited a cohort of 184 adolescent patients with depression and the same number of healthy controls with similar ages to investigate the possible etiology of adolescent depression. Intriguingly, we did not find any significant difference in the levels of inflammatory markers hs-CRP or TNF-α between the patient and control groups. However, significantly higher serum levels of histamine, S100B, and homocysteine, as well as a higher positive rate of serum food-specific IgG, were demonstrated in the patient group compared to the control group. A higher level of histamine suggests a hypersensitivity mediated by food antigen-specific IgG, which together with the enhanced levels of S100B indicates that the BBB leakage may play a potential role in the occurrence and development of adolescent depression. Therefore, the concept of chronic food antigen-specific IgG-mediated hypersensitivity or chronic food intolerance, rather than the chronic low-grade inflammation, should be acknowledged or emphasized in the pathogenesis of adolescent depression.

An increased serum histamine level has been shown to lead to a higher BBB permeability [18], which is also reflected by the increased level of S100B even without brain damage [28], [30]. Histamine normally can’t pass through BBB, however, the histamine-induced increase in BBB permeability could, in turn, promote the entry of histamine into the CNS. Such a concept is supported by previous reports demonstrating higher histamine levels in the cerebrospinal fluid and brain parenchyma of individuals with various neurodegenerative diseases [4], [5], [6]. Additionally, histamine can increase the production of some proinflammatory cytokines including TNF-α, nitric oxide (NO), and reactive oxygen species (ROS), leading to mitochondrial membrane potential dysfunction [31]. Based on these studies and our findings, it is reasonable to postulate that higher blood histamine levels are closely associated with the onset of adolescent depression.

The development of neuroinflammation could be explained via a number of mechanisms. First, the significantly higher positive rate of food antigen-specific IgG found in the patient group suggests an increased ability of food antigens to enter the systemic circulation and form an immune complex with food antigen-specific IgG. While the large complexes can be easily removed by macrophages, the small ones are very difficult to remove [32]. Therefore, these small complexes easily enter the CNS, triggering neuroinflammation, or precipitate and become deposited in blood vessels, joints, glomeruli, and other tissues, causing tissue damage, inflammation, and metabolic abnormalities [33]. Some evidence has shown that the high permeability and neurovascular dysfunction of the BBB are closely related to MDD [19]. Second, the high intestinal wall permeability caused by food antigen-specific IgG-mediated hypersensitivity reaction can cause endotoxins from intestinal Gram-negative bacteria to enter the systemic circulation and the CNS under conditions of high BBB permeability, and this process can lead to local cerebral neuroinflammation [3]. Third, the higher serum homocysteine levels may interact with the *N*-methyl-D-aspartate receptor to produce free radicals that induce neurotoxicity [30]. In addition, our previous study revealed elevated serum uric acid level in adolescent patients with depression [34]. All of these factors can enter the CNS and cause cell damage and inflammatory response through the enhanced oxidative stress under the condition of high BBB permeability. Moreover, once these factors enter the CNS through the highly permeable BBB, they stimulate astrocytes and microglia to produce proinflammatory cytokines. These proinflammatory cytokines affect the metabolic processes of certain neurotransmitters, such as 5-HT and dopamine, thereby affecting neuronal transmission.

Focusing on first-episode depression patients to explore the etiology of adolescent depression has an advantage over focusing on adult patients with depressive disorders because many factors, such as chronic diseases, substance addiction, and social environment, may interfere with clinical outcomes. The cohort that was evaluated in our current study included only students aged 14–20 years with similar social environments and also exclusion of any chronic diseases. All the patients were first diagnosed without receiving any antidepressant treatment before the clinical study. Therefore, our findings should accurately reflect the early changes in biological markers related to depression. More importantly, our findings suggest that the pathogenesis of adolescent depression is different from that of adult depression, with systemic inflammation playing a role in the disease process. Therefore, our results may suggest a stage-specific manifestation evidenced by the increased serum uric acid level found in adolescents with depression in our previous report [34]. Moreover, adult depression often originates from the onset of adolescent depression [17].

## Conclusion

According to our studies, we believe that adolescent depression may result from immune disorders, metabolic disorders, and nutritional imbalances. These findings suggest that to block food antigen-specific IgG-mediated hypersensitivity may be a new mechanism for the treatment of MDD. Therefore, avoiding allergy-inducing food and using antihistamine drugs may be the future direction of adolescent depression treatment, and these methods have been included in our future research plan. Second, the detection of IgG, IgE, histamine, and other indicators would provide a new objective basis for the early diagnosis of depression, and also provide a reliable basis for the evaluation of the treatment of depression. Finally, in addition to MDD, other CNS diseases such as Alzheimer's disease, Parkinson's disease, and epilepsy are also associated with increased BBB permeability. Therefore, we conclude that long-term food antigen-specific IgG-mediated hypersensitivity may also be associated with the pathogenesis of these CNS diseases, and this topic remains worthy of further investigations.

## Materials and methods

### Subject information

#### Patient enrollment for the ADP group

Patient enrollment was performed by a chief physician, an attending physician, and two residents from the Department of Addiction Medicine, the Seventh Medical Center of the General Hospital of the People's Liberation Army (PLAGH).

Patients first visited the Department from February 2015 to December 2016 from all over China. The inclusion criteria for patient enrollment are as follows. (1) Patients met the diagnostic criteria for depression according to the Diagnostic and Statistical Manual of Mental Disorders, volume 5 (DSM-5). (2) Patients had a HAMD-17 score ranging 17–24. (3) Patients were aged 14–20 years. (4) Patients were not treated with any psychotropic drugs. (5) The patients were free diseases of immune system, liver, and kidney, gout, or any physical diseases. (6) Patients had normal levels of blood urea nitrogen and creatinine.

#### Control enrollment for the NAS group

Control enrollment was completed by an attending psychiatrist and two residents from the Department of Addiction Medicine, the Seventh Medical Center of the PLAGH. The healthy controls were Beijing high school students who came to the hospital for a medical checkup from February 2015 to December 2016. Physical examinations indicated that these students were healthy. In addition, these students did not have any mental disorders based on a psychiatric examination by the attending psychiatrist.

Both patients and control individuals answered the HAMD-17 questionnaire and read and signed the informed consent form that was reviewed and approved by the Ethical Review Committee of the Seventh Medical Center, PLAGH.

### Blood sample collection

Blood samples from the patient group were collected by the Department of Addiction Medicine, the Seventh Medical Center of the PLAGH within 3 days of patient admission, while blood samples from the control group were collected on the day of medical check-up. All subjects were forbidden to smoke, drink or use various drugs three days before testing and consumed a light diet. For each subject, 4 ml of fasting blood samples were collected in the morning into blood collection tubes and agglutinated at the room temperature. Serum was centrifuged at 3000 rpm for 10 min at 4 °C and the resulting supernatant was stored at –20 °C or –80 °C prior to use.

### Biochemical analyses

All blood samples were sent to Di’an Medical Examination Center (Beijing, China) for testing.

#### Measurement of CRP (hs-CRP), IgE, and TNF-α concentrations

An emulsion technique and electrochemiluminescence were adopted for the measurement of CRP and IgE using the c701 and e602 modules, respectively, on an automatic biochemical analyzer (Catalog No Cobas8000; Roche, Germany), with the matching original reagents (Catalog Nos 05950864190 [35] and 04827031190 [36]), respectively. The concentration of TNF-α was measured chemiluminescently on an immunoassay system (Immulite1000, Catalog No. 04827031190; Siemens) [35].

#### Measurement of food antigen-specific IgG concentrations

An ELISA assay was used to simultaneously determine the levels of 14 food-specific IgG antibodies in the serum samples obtained from all subjects, following the manufacturer’s instructions (Catalog No. 7194; Biomerica; Irvine, CA) [36]. The food-specific IgG antibodies tested include milk, beef, chicken, pork, codfish, corn, rice, crab, egg white/yolk, shrimp, soybean, tomato, mushroom, and wheat.

#### Measurement of homocysteine, histamine, S100B, and DAO concentrations

Enzymatic cycling assay was used to measure homocysteine on an automatic biochemical analyzer (Catalog No Cobas8000; Roche, Germany) using the c701 module and the matching original reagent (Catalog No. C0805) [37]. Concentration of histamine, S100B, and DAO was measured by ELISA, double-antibody sandwich ELISA, and micromethod using the reagent kit from Novus Biologicals (Catalog No. NBP2-62860), R&D Systems (Catalog No. DY1820-05), and Shanghai Yubo Biotechnology (Catalog No. YB-DAO-Hu), respectively. All the assays were measured on an A0018T Microplate reader (Thermo-Fisher Scientific, USA) following the instructions of reagent suppliers [38], [39].

### Statistical analysis

Data were presented as number and percentages for categorical variables, while continuous data of normal distribution was expressed as mean ± standard deviation (SD) and continuous data of non-normal distribution were expressed as median (quartile spacing) [M (P25, P75)], and Mann–Whitney’s *U* test was used for comparison between groups. Inter-group difference was compared using Chi-square test or Fisher’s exact test for categorical variables (gender, distribution of patients positive for TNF-α, hs-CRP, IgE, and food antigen-specific IgG). All statistical analyses were performed using GraphPad Prism 6.0 (GraphPad Software, San Diego, CA), where *P* < 0.05 was considered statistically significant.

## Authors’ contributions

RT conceived the idea and designed the project. ZF and LX analyzed the data. ZF and LX drafted the manuscript. All authors edited the manuscript, read and approved the final manuscript.

## Competing interests

The authors declare no conflicts of interest.
